# Bronchus compression is a predictor for reobstruction in coarctation with hypoplastic arch repair

**DOI:** 10.1093/icvts/ivad186

**Published:** 2023-11-22

**Authors:** Chengyi Hui, Qiushi Ren, Jian Zhuang, Jimei Chen, Xiaohua Li, Hujun Cui, Jianzheng Cen, Gang Xu, Shusheng Wen

**Affiliations:** Department of Cardiac Surgery, Guangdong Provincial People's Hospital (Guangdong Academy of Medical Sciences), Southern Medical University, Guangzhou, China; Department of Cardiac Surgery, Guangdong Provincial People's Hospital (Guangdong Academy of Medical Sciences), Southern Medical University, Guangzhou, China; Department of Cardiac Surgery, School of Medicine, South China University of Technology, Guangzhou, China; Department of Cardiac Surgery, Guangdong Provincial People's Hospital (Guangdong Academy of Medical Sciences), Southern Medical University, Guangzhou, China; Department of Cardiac Surgery, School of Medicine, South China University of Technology, Guangzhou, China; Department of Cardiac Surgery, Guangdong Provincial People's Hospital (Guangdong Academy of Medical Sciences), Southern Medical University, Guangzhou, China; Department of Cardiac Surgery, Guangdong Provincial People's Hospital (Guangdong Academy of Medical Sciences), Southern Medical University, Guangzhou, China; Department of Cardiac Surgery, Guangdong Provincial People's Hospital (Guangdong Academy of Medical Sciences), Southern Medical University, Guangzhou, China; Department of Cardiac Surgery, Guangdong Provincial People's Hospital (Guangdong Academy of Medical Sciences), Southern Medical University, Guangzhou, China; Department of Cardiac Surgery, Guangdong Provincial People's Hospital (Guangdong Academy of Medical Sciences), Southern Medical University, Guangzhou, China; Department of Cardiac Surgery, Guangdong Provincial People's Hospital (Guangdong Academy of Medical Sciences), Southern Medical University, Guangzhou, China

**Keywords:** Coarctation of aorta, Hypoplastic aortic arch, Aortic arch geometry, Airway compression, Arch reobstruction

## Abstract

**OBJECTIVES:**

The surgical treatment of coarctation of aorta with hypoplastic aortic arch (CoA/HAA) was challenging to achieve long-lasting arch patency. We reviewed early and late outcomes in our centre and identified predictors for arch reobstruction.

**METHODS:**

A retrospective analysis of medical records was performed to identify CoA/HAA patients who underwent primary arch reconstruction via median sternotomy between 2011 and 2020. Preoperative aortic arch geometry was analysed with cardiac computed tomographic angiography. Bedside flexible fibre-optic bronchoscopy was routinely performed after surgery in intensive care unit.

**RESULTS:**

There were 104 consecutive patients (median age 39.5 days) who underwent extended end-to-end anastomosis, extended end-to-side anastomosis and autograft patch augmentation. Early mortality was 3.8% and overall survival was 94.1% [95% confidence interval (CI) 89.6–98.8%] at 1, 3 and 5 years. Reobstruction-free survival was 85.1% (95% CI 78.4–92.3%) at 1 year, 80.6% (95% CI 73.1–88.9%) at 3 years and 77.4% (95% CI 69.2–86.6%) at 5 years. Preoperative aortic arch geometric parameters were not important factors for reobstruction. Nineteen patients (18.3%) were detected with left main bronchus compression (LMBC) on flexible fibre-optic bronchoscopy. Cardiopulmonary bypass time [*P* < 0.001, hazard ratio (95% CI): 1.02 (1.01–1.03)] and postoperative LMBC [*P* = 0.034, hazard ratio (95% CI): 2.99 (1.09–8.23)] were independent predictive factors on multivariable Cox regression analysis of reobstruction-free survival.

**CONCLUSIONS:**

Aortic arch can be satisfactorily repaired by extended end-to-end anastomosis, extended end-to-side anastomosis and autograft patch augmentation via median sternotomy in CoA/HAA. Cardiopulmonary bypass time and postoperative LMBC detected by flexible fibre-optic bronchoscopy are significant predictors for long-term arch reobstruction.

**Figure:**
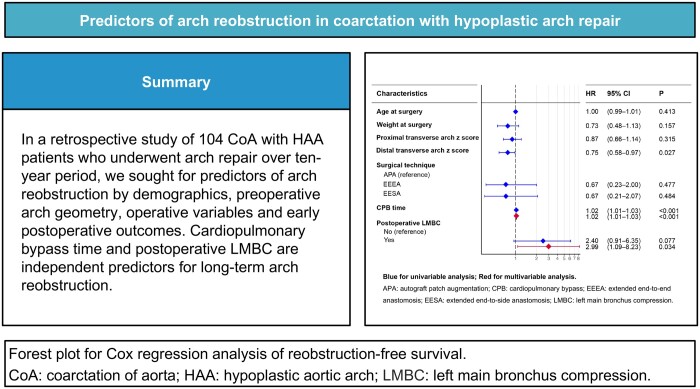

## INTRODUCTION

Hypoplastic aortic arch (HAA) is commonly comorbid with coarctation of aorta (CoA) in neonates and infants. Due to the varied definitions of HAA in the literature, the incidence of HAA in CoA patients ranged from 14% to 31% [1–3], even up to 93% in those with additional intracardiac anomalies [4]. Various surgical techniques, including extended end-to-end anastomosis (EEEA), extended end-to-side anastomosis (EESA) and pericardial/pulmonary autograft patch augmentation (APA), have been proposed to deal with changeful transverse aortic arch hypoplasia. An optimal repair should be associated with low mortality, low morbidity and low reobstruction/reintervention rate. However, there is still lack of long-term follow-up data to make an optimal choice for different arch geometry.

The morphology of an aortic arch is of great significance to the normal circulatory physiology. Although multiple techniques and manners are applied in arch reconstruction to modify a ‘successful’ shape, the deterministic factors in the shape features of an aortic arch late after repair are essentially intrinsic or inherently altered [5]. Several investigators identified the preoperative arch size and some specific arch anatomies were related to reobstruction/reintervention [6–8]. But a conclusive association between the preoperative aortic arch geometry and clinical outcomes remained elusive. Several investigations figured out the tissue-to-tissue arch repair technique could result in real airway problems [9, 10]. However, the incidence of airway compression after aortic arch reconstruction varies greatly in different literatures [9, 11, 12]. The lack of awareness in postoperative airway problems and the lack of effective airway assessment method may account for this difference. Early airway compression detection and management may reduce further morbidity [10]. Thus, we propose the aortic arch geometry study and early postoperative airway assessment may be valuable in predicting arch reobstruction after CoA with HAA (CoA/HAA) repair.

This study aims to (i) determine the early and late outcomes after CoA/HAA repairs in our centre and (ii) investigate effects of preoperative aortic arch geometry and postoperative airway compression problems on long-term arch reobstruction.

## MATERIALS AND METHODS

### Ethics statement

This study was approved by Guangdong Provincial People's Hospital Ethics Committee (registration number GDREC2019338H(R2)) on September 17, 2019. Individual consent was waived due to the retrospective nature.

### Study population and design

We retrospectively reviewed the medical records of patients who had undergone primary aortic coarctation repairs via median sternotomy at Paediatric Cardiac Surgery Department, Guangdong Provincial People's Hospital between January 2011 and December 2020. Both transthoracic echocardiography (TTE) and cardiac computed tomographic angiography were performed for CoA diagnosis and aorta evaluation before surgery. HAA was diagnosed when a *z* score of transverse (proximal or distal) aortic arch was <−2. *z* scores were calculated using Pettersen’s regression model [13] by cardiac computed tomographic angiography. Patients with severe aortic or subaortic stenosis (requiring Norwood–Rastelli procedures) or single ventricle were excluded. Early outcomes were defined as those occurred within 30 postoperative days. Late outcomes occurred after this period of time. All patients were recommended to visit the outpatient clinic and receive TTE every 6–12 months after discharge. Follow-up was performed by reviewing the outpatient charts or contacting patients. Arch reobstruction was determined by TTE reports with a maximum pressure gradient across the anastomotic site ≥25 mmHg. The primary outcome of interest was duration of reobstruction-free survival, defined as the time from arch repair to reobstruction or death.

### Operative strategy and technique

All patients underwent one-stage repair for aortic reconstruction and intracardiac defect correction (if concomitant). We reconstructed aortic arch with EEEA, EESA or APA via median sternotomy under cardiopulmonary bypass (CPB). Both deep hypothermic circulatory arrest (DHCA) and antegrade cerebral perfusion (ACP) strategies were used.

After median sternotomy performed, the aortic aorta and its branches were fully mobilized, and the descending aorta was extensively dissected to the level of the third intercostal arteries. After heparinization, a straight-tip arterial cannula was inserted in the ascending aorta, just below the emergence of the innominate artery. Venous drainage was obtained by bicaval cannulation. Supplementary ductal arterial cannulation is performed in patients with significant CoA in which poor distal perfusion may happen. The patent ductus arteriosus or ligamentum arteriosum was ligated. After cooling to 32°C, the aorta was clamped and cold crystalloid cardioplegia was administered in the aortic root. In the following we demonstrate surgical procedures under ACP. Concomitant intracardiac corrections were performed while the temperature was reduced to 25°C. The arterial cannula was advanced in the innominate artery and snared in position. A period of ACP was commenced. All ductal tissue was removed. In EEEA, an incision was made in the inner curve of the arch close to the distal transverse arch (Supplementary Material, Fig. S1). In EESA, an incision was made in the under surface of the distal ascending aorta and proximal transverse arch, usually opposite to the emergence of the innominate artery (Supplementary Material, Fig. S1). The descending aorta incised laterally to extend was directly anastomosed to the opening of the arch in both EEEA and EESA. In APA, an incision was made in the inner curve of the arch extended from the distal ascending aorta to the distal transverse arch (Supplementary Material, Fig. S1). A 10 mm × 20 mm autograft patch was harvested from the anterior wall of the main pulmonary artery (then repaired with a same-size fresh autologous pericardial patch) or from the fresh autologous pericardium. The distal aorta was anastomosed to the aortic arch to form a native outer curve and the patch was sutured to augment the anterior and inferior wall of the arch. The anastomosis was performed with a running polypropylene suture.

### Flexible fibre-optic bronchoscopy

Our centre had performed an airway assessment protocol for CoA patients since 2010: bedside flexible fibre-optic bronchoscopy was performed by a trained paediatric critical care physician using a validated endoscope (LF-TP, LF-DP; Olympus Corp, Tokyo, Japan) with the use of sedatives 24–48 h after surgery under intubation in cardiac intensive care unit (ICU). Airway compression was defined as the indentation of the trachea and bronchi during exhalation, and local pulsatile signs that varied with heart rate could be seen. The degree was defined by the ratio of lumen compression: mild <1/2, moderate 1/2 to 3/4 and severe >3/4.

### Statistical analysis

Statistical analyses were performed using software SPSS 26 (IBM Corp., Armonk, NY), R studio (Version 4.1.2, www.r-project.org) and GraphPad Prism 8.4 (GraphPad Software Inc, San Diego, Calif). Continuous variables were presented as median with interquartile range (IQR) or as mean ± standard deviation. Descriptive variables were presented as absolute numbers and percentages for categorical variables. To compare patients who underwent EEEA, EESA and APA, we used single-factor analysis of variance or Kruskal–Wallis *H* test for continuous variables and chi-squared test for categorical variables. To compare patients with and without postoperative left main bronchus compression (LMBC), we used unpaired *t*-test or Mann–Whitney *U*-test for continuous variables and chi-squared test for categorical variables. Reobstruction-free survival was analysed by Kaplan–Meier curve analysis using Mantel–Cox log-rank test. The Cox proportional hazard regression model was used to assess the association between 1 or more continuous or categorical variables and the time of onset of reobstruction or death. Variables with a *P*-value of <0.1 on univariable analysis, including distal transverse arch *z* score, CPB time, aortic cross-clamp (ACC) time and postoperative LMBC, were entered into a stepwise multivariable Cox regression analysis. A *P*-value of <0.05 was accepted as a statistically significant difference. No adjustments were conducted in multiple comparisons, so *P*-values may not be interpreted as confirmatory but rather descriptive.

## RESULTS

### Patient characteristic and preoperative aortic arch geometry

A total of 104 patients were included. The median age at surgery was 39.5 days (IQR 14.2–80.7 days), and the oldest patient was 51 months old. The numbers of neonates and infants were 41 (39.4%) and 98 (94.2%), respectively. The median weight at surgery was 3.70 kg (IQR 3.30–4.50 kg), and 6 patients (5.8%) weighed <2.5 kg. According to the surgical techniques for aortic reconstruction, patients were divided into 3 cohorts: the EEEA group, 41 patients; the EESA group, 38 patients; the APA group, 25 patients (Table 1). In the APA group, only 2 patients underwent autologous pericardial patch augmentation, while 23 patients underwent autologous pulmonary patch augmentation. The EEEA group had larger median *z* scores and shorter median length both in the proximal and distal transverse arch than the other 2 groups. The median length of both proximal and distal transverse in the EESA group was the largest among the 3 groups. The APA group had smaller median *z* scores of the proximal transverse arch, the distal transverse arch and the aortic isthmus than the other 2 groups. All aortic arch geometric parameters among the 3 groups were not statistically different.

**Table 1:** Patient characteristics and preoperative aortic arch geometry

Variable	Overall (*n* = 104)	EEEA (*n* = 41)	EESA (*n* = 38)	APA (*n* = 25^a^)	*P*-value
Male gender, *n* (%)	66 (63.5)	26 (63.4)	26 (68.4)	14 (56.0)	0.606
Age at surgery (days), median (IQR)	39.5 (14.2 to 80.7)	40.0 (13.5 to 66.5)	26.5 (13.7 to 98.5)	48.0 (23.0 to 72.5)	0.750
Weight at surgery (kg), median (IQR)	3.70 (3.30 to 4.50)	3.70 (3.20 to 4.52)	3.75 (3.30 to 4.75)	3.70 (3.15 to 4.05)	0.793
Preoperative PGE1 use, *n* (%)	17 (16.3)	7 (17.1)	7 (18.4)	3 (12.0)	0.786
Preoperative mechanical ventilation support, *n* (%)	46 (44.2)	16 (39.0)	23 (60.5)	7 (28.0)	0.027
Proximal transverse arch *z* score, median (IQR)	−2.57 (−3.80 to −1.93)	−2.41 (−3.93 to −1.81)	−2.50 (−4.05 to −1.91)	−2.74 (−3.53 to −2.15)	0.813
Proximal transverse arch length (mm), median (IQR)	2.1 (0 to 3.0)	2.0 (0 to 2.7)	2.5 (0 to 3.5)	2.1 (0.4 to 2.9)	0.760
Distal transverse arch *z* score, median (IQR)	−3.10 (−4.05 to −2.38)	−2.94 (−3.84 to −2.27)	−3.40 (−4.37 to −2.20)	−3.50 (−4.40 to −2.50)	0.450
Distal transverse arch length (mm), median (IQR)	6.1 (3.5 to 8.3)	5.6 (3.2 to 8.9)	6.2 (4.0 to 7.9)	6.1 (3.4 to 7.2)	0.750
Aortic isthmic *z* score, median (IQR)	−3.88 (−5.01 to −2.69)	−3.73 (−5.08 to −2.93)	−3.62 (−5.00 to −2.40)	−4.27 (−5.19 to −2.93)	0.661
Bovine aortic arch, *n* (%)	29 (27.9)	11 (26.8)	12 (31.6)	6 (24.0)	0.791
Aberrant right subclavian artery, *n* (%)	3 (2.9)	1 (2.4)	0 (0)	2 (8.0)	0.174

a In the APA group, only 2 patients underwent autologous pericardial patch augmentation, while 23 patients underwent autologous pulmonary patch augmentation.

APA: autograft patch augmentation; EEEA: extended end-to-end anastomosis; EESA: extended end-to-side anastomosis; IQR: interquartile range.

### Operative data

CPB time and ACC time in the APA group were 182 ± 58 and 115 ± 33 min (Table 2), both of which were significantly longer than those in the EEEA and EESA groups (CPB time, *P* = 0.001; ACC time, *P* < 0.001). The ACP strategy accounted for 76.0% in the APA group, which was significantly higher (*P* < 0.001) than that in the EEEA group (24.4%) and the EESA group (42.1%). DHCA time and ACP time in the APA group were significantly longer than those in the other 2 groups (DHCA time, *P* < 0.001; ACP time, *P* < 0.001). The most common concomitant procedures were atrial septal defect closure (85.6%) and ventricular septal defect closure (83.7%) (Supplementary Material, Table S1).

**Table 2:** Operative characteristics

Variable	Overall (*n* = 104)	EEEA (*n* = 41)	EESA (*n* = 38)	APA (*n* = 25)	*P*-value
CPB time (min), mean ± SD	150 ± 52	138 ± 51	142 ± 39	182 ± 58	0.001
ACC time, min, mean ± SD	74 ± 34	59 ± 21	64 ± 22	115 ± 33	<0.001
DHCA, *n* (%)	59 (56.7)	31 (75.6)	22 (57.9)	6 (24.0)	<0.001
DHCA time (min), mean ± SD	19 ± 6	18 ± 5	18 ± 4	32 ± 5	<0.001
ACP, *n* (%)	45 (43.3)	10 (24.4)	16 (42.1)	19 (76.0)	<0.001
ACP time (min), mean ± SD	41 ± 19	30 ± 12	27 ± 10	58 ± 14	<0.001

ACC: aortic cross-clamp; ACP: antegrade cerebral perfusion; APA: autograft patch augmentation; CPB: cardiopulmonary bypass; DHCA: deep hypothermic circulatory arrest; EEEA: extended end-to-end anastomosis; EESA: extended end-to-side anastomosis; SD: standard deviation.

### Early outcomes

All patients underwent bedside TTE in ICU after surgery, and only 2 patients had anastomotic pressure gradient ≥25 mmHg (one in the EEEA group 38 mmHg and the other in the EESA group 25 mmHg). There was no aortic reintervention within 30 postoperative days. The most common early postoperative adverse events (Table 3) requiring medical management were wound adverse events (including wound dehiscence and superficial wound infection). All the 12 wound adverse events occurred in patients who experienced delayed sternal closure. Two patients (1.9%), one each in the EEEA group and the EESA group, developed severe low cardiac output syndrome within 24 h after surgery and had to undergo extracorporeal membrane oxygenation.

**Table 3:** Early outcomes

Variable	Overall (*n* = 104)	EEEA (*n* = 41)	EESA (*n* = 38)	APA (*n* = 25)
Postoperative mechanical ventilation time (h), median (IQR)	104.5 (73.5 to 149.5)	120.5 (74.5 to 168.0)	97.5 (68.1 to 140.1)	97.5 (83.8 to 146.9)
Postoperative ICU length of stay (days), median (IQR)	16.0 (11.0 to 20.0)	15.0 (9.5 to 20.0)	16.5 (9.8 to 21.3)	16.5 (11.5 to 20.8)
Delayed sternal closure, *n* (%)	31 (29.8)	16 (39.0)	8 (21.1)	7 (28.0)
Adverse events, *n* (%)				
Unplanned mediastinal exploration	3 (2.9)	3 (7.3)	0 (0)	0 (0)
Neurologic events	4 (3.8)	1 (2.4)	2 (5.3)	1 (4.0)
ECMO	2 (1.9)	1 (2.4)	1 (2.6)	0 (0)
Wound adverse events	12 (11.5)	4 (9.8)	4 (10.5)	4 (16.0)
Diaphragm paralysis	5 (4.8)	1 (2.4)	3 (7.9)	1 (4.0)
Left main bronchus compression	19 (18.3)	9 (22.0)	7 (18.4)	3 (12.0)
Early mortality	4 (3.8)	1 (2.4)	1 (2.6)	2 (8.0)

APA: autograft patch augmentation; ECMO: extracorporeal membrane oxygenation; EEEA: extended end-to-end anastomosis; EESA: extended end-to-side anastomosis; ICU: intensive care unit; IQR: interquartile range.

**Figure 1:**
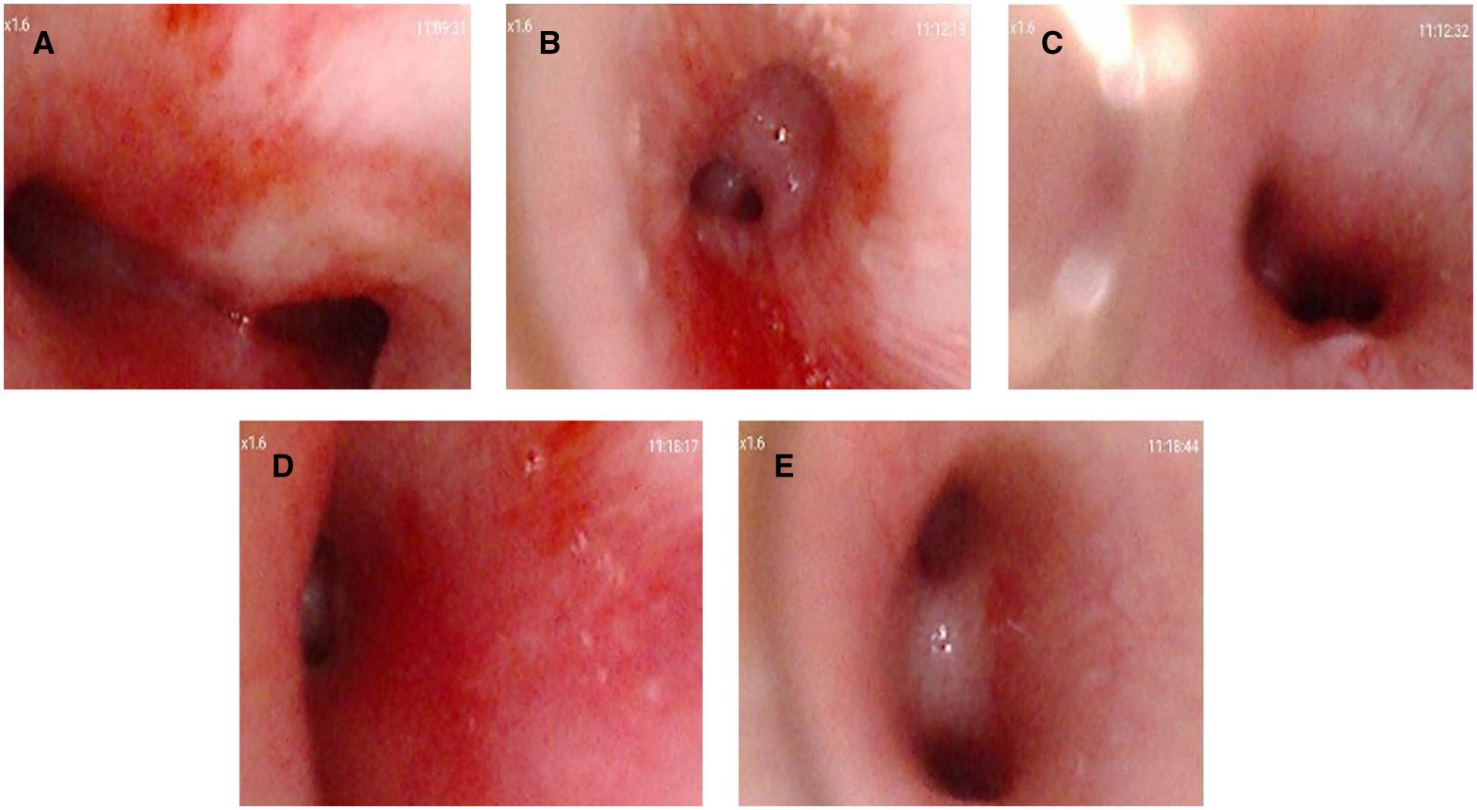
The images in flexible fibre-optic bronchoscopy from a patient with left main bronchus compression. (**A**) Normal trachea carina. (**B**) Normal right upper lobe bronchus. (**C**) Normal right middle bronchus. (**D**) Mild compressed left main bronchus. (**E**) Normal upper and lower left lobe bronchi.

A total of 19 patients (18.3%) were detected postoperative LMBC on flexible fibre-optic bronchoscopy. Nine patients (22.0%) in the EEEA group, 7 patients (18.4%) in the EESA group and 3 patients (12.0%) in the APA group experienced LMBC. All compression in the left main bronchus was considered mild (Fig. 1). Four of these 19 patients developed left pulmonary atelectasis and 7 suffered respiratory distress after extubating. No additional surgical procedures were performed to relieve airway compression. The patients with LMBC had significantly lower median body weight (*P* = 0.046) and significantly higher proportion of preoperative PGE1 use (*P* = 0.008) than patients without (Supplementary Material, Table S2). The patients with LMBC suffered significantly longer postoperative ICU length of stay (*P* = 0.037) and hospital length of stay (*P* = 0.002) than patients without.

Early mortality was 3.8% (*n* = 4). One died from complications related to extracorporeal membrane oxygenation. One died of iatrogenic postoperative third-degree A–V block. The other 2 experienced left ventricular dysfunction and were barely responsive to medical management, eventually withdrawing support.

### Late outcomes

**Figure 2:**
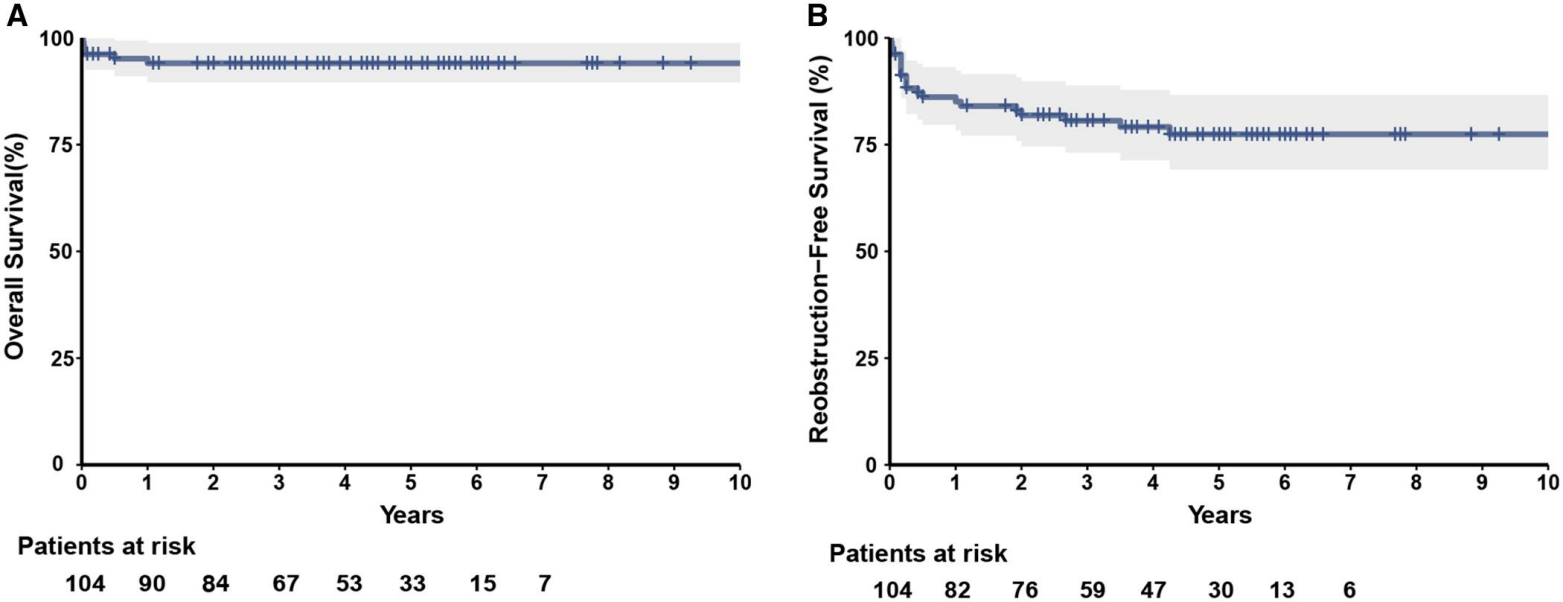
Kaplan–Meier curves depicting overall survival (**A**) and reobstruction-free survival (**B**). The shaded area represents the 95% confidence intervals.

Follow-up was available from 100 patients with a median follow-up duration of 4.3 years (IQR 2.6–5.6 years). There were 2 late deaths (2.0%). One death was attributed to a cardiac arrest presumably due to a life-threatening arrhythmia 6 months after surgery. The cause of another death which occurred 1 year after surgery was unknown. Both deaths occurred in the APA group without positive findings in flexible fibre-optic bronchoscopy. Overall survival was 94.1% [95% confidence interval (CI) 89.6–98.8%] at 1, 3 and 5 years (Fig. 2A). Four patients underwent 5 cardiac reinterventions during the follow-up. Only one of 5 reinterventions involved aortic reobstruction, which occurred within 1 year after initial repair. Two patients underwent aortic subvalvular myectomy. Two patients developed pulmonary stenosis: one underwent surgical patch augmentation repair and the other had successful balloon angioplasty.

**Figure 3:**
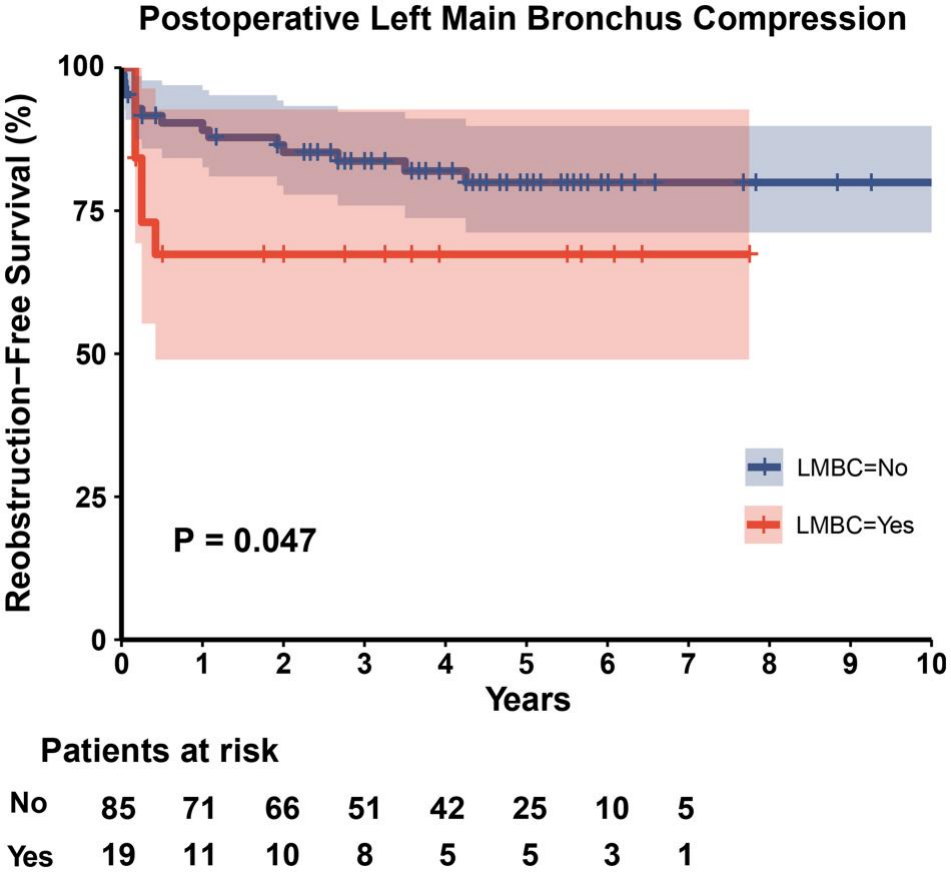
Kaplan–Meier curves depicting reobstruction-free survival for patients with or without postoperative left main bronchus compression. The shaded area represents the 95% confidence intervals. The Mantel–Cox log-rank test showed a significant difference in reobstruction-free survival between the 2 groups (*P* = 0.047).

Fifteen patients had echocardiographic evidence of arch reobstruction. Six patients (15.0%) in the EEEA group, 5 patients (13.5%) in the EESA group and 4 patients (17.4%) in the APA group experienced reobstruction. These gradients across arch anatomies in 1 patient rapidly increased and surgical reinterventions were performed during the follow-up period. Reobstruction-free survival was 85.1% (95% CI 78.4–92.3%) at 1 year, 80.6% (95% CI 73.1–88.9%) at 3 years and 77.4% (95% CI 69.2–86.6%) at 5 years (Fig. 2B). With the use of the Mantel–Cox log-rank test, there was no significant difference in reobstruction-free survival among the 3 groups (*P* = 0.33) (Supplementary Material, Fig. S2). CPB time [*P* < 0.001, hazard ratio (95% CI): 1.02 (1.01–1.03)] and postoperative LMBC [*P* = 0.034, hazard ratio (95% CI): 2.99 (1.09–8.23)] were independent predictive factors on multivariable Cox regression analysis of reobstruction-free survival (Table 4). The Mantel–Cox log-rank test showed significant difference in reobstruction-free survival between patients with and without LMBC (*P* = 0.047) (Fig. 3).

**Table 4:** Cox proportional hazard regression analysis of reobstruction-free survival

Variable	Univariable analysis		Multivariable analysis	
	HR (95% CI)	*P*-value	HR (95% CI)	*P*-valve
Age at surgery	1.00 (0.99–1.01)	0.413		
Weight at surgery	0.73 (0.48–1.13)	0.157		
Preoperative PGE1 use	2.27 (0.81–6.35)	0.117		
Preoperative mechanical ventilation support	1.90 (0.76–4.74)	0.168		
Proximal transverse arch *z* score	0.87 (0.66–1.14)	0.315		
Proximal transverse arch length	1.00 (0.78–1.28)	0.983		
Distal transverse arch *z* score	0.75 (0.58–0.97)	0.027		
Distal transverse arch length	1.04 (0.92–1.18)	0.538		
Aortic isthmic *z* score	0.93 (0.74–1.18)	0.554		
Bovine aortic arch	0.97 (0.35–2.69)	0.952		
Surgical technique				
APA	–	–		
EEEA	0.67 (0.23–2.00)	0.477		
EESA	0.67 (0.21–2.07)	0.484		
CPB time	1.02 (1.01–1.03)	<0.001	1.02 (1.01–1.03)	<0.001
ACC time	1.02 (1.01–1.03)	0.001		
Postoperative mechanical ventilation time	1.00 (0.99–1.01)	0.754		
Postoperative left main bronchus compression	2.40 (0.91–6.35)	0.077	2.99 (1.09–8.23)	0.034

ACC: aortic cross-clamp; APA: autograft patch augmentation; CI: confidence interval; CPB: cardiopulmonary bypass; EEEA: extended end-to-end anastomosis; EESA: extended end-to-side anastomosis; HR: hazard ratio.

## DISCUSSION

The incidence of airway compression after arch repairs varies widely among different studies. Based on clinical manifestation for diagnosis, the incidence of LMBC was 0.7–2.2% [11, 12, 14]. However, depending on cardiac CT and intraoperative flexible bronchoscopy for airway assessment and management, Jhang *et al.* [9] identified airway compression in 23 of 90 patients (25.6%) after arch reconstruction, which was a rather high incidence. In our study, with routine use of a flexible fibre-optic bronchoscope after surgery, the incidence (18.3%) was lower than that in Jhang's study. A normal aortic arch produces a wide and natural curve and encircles the origin of the right pulmonary artery and the left main bronchus. An arch repair, especially by the tissue-to-tissue technique, can cause shortening of the space between ascending and descending aorta and result in LMBC. In this study, we found that LMBC and CPB were independent predictors for long-term arch reobstruction, both of which were for sure not the causes. For LMBC, the rational explanation for this association is not enough distance between the parts of aorta causing the compression and at the same time the tension on the anastomosis causing the scar tissue development and restenosis in the late follow-up. As for CPB, it may be a consequence of the technical problems, like necessity of the revision of the anastomosis or the intraoperative bleeding and need for additional haemostatic sutures. In our research, none of the 19 patients with LMBC required reinterventions to relieve the compression, but they did require longer duration of ventilator support, ICU stay and hospital stay, which meant heavier health economics burden. In our centre, it had become a routine to perform bedside flexible fibre-optic bronchoscopy after surgeries involving aorta/vascular ring/airway in ICU. We recommend meticulous airway assessment by routine flexible fibre-optic bronchoscopy early after arch repair. This may change our patients’ care. Routine flexible fibre-optic bronchoscopy can early and clearly detect airway problems. For severe airway compression caused by failed surgical anastomosis, reinterventions could be performed timely. For mild conditions, we could carefully evaluate the indications for extubating, strengthen airway management and avoid reintubation. After discharge, we would strengthen patient education and pay more follow-up attention to patients with LMBC.

Several studies described the effects of preoperative aortic arch geometry on clinical outcomes. Tulzer *et al.* [6] assessed 183 CoA/HAA infants who underwent aortic arch reconstruction via thoracotomy and sternotomy approaches. They identified a *z* score of proximal transverse arch of −4.59 with a sensitivity of 100% and a specificity of 98.77% as a cut-off value in terms of prediction on reintervention. Interestingly, a total of 11 patients underwent aortic reintervention during their follow-up, and 10 of these 11 patients underwent primary aortic reconstruction with EEEA via thoracotomy. Kaushal *et al.* [7] assessed 201 CoA patients (157 patients via thoracotomy and 44 patients via sternotomy) who underwent EEEA and found aberrant right subclavian artery was the only risk factor for reobstruction in univariable Cox regression analysis. Turek *et al.* [8] investigated 49 patients underwent EEEA via thoracotomy and found children with bovine arch anatomy were at a significantly increased risk of reobstruction. These findings inspired us to explore the influence of preoperative aortic arch geometry on clinical outcomes. However, in our study, all patients underwent aortic repair via median sternotomy, and no statistical significance was found in the proposed preoperative arch geometry on univariable and multivariable Cox regression analysis. A possible explanation lies in the effect of surgical approach choice rather than a specific preoperative aortic arch geometry on outcomes. For patients with CoA/HAA, median sternotomy is more conducive to fully mobilize the ascending aorta and its branches than lateral thoracotomy. This is very important to ensure the reconstructed arch free from distortion and high tension. Under these preconditions, an appropriate surgical technique can always achieve satisfactory outcomes when facing severe hypoplastic arch, bovine arch, or aberrant right subclavian artery.

Aortic arch reobstruction is the main complication after a primary arch repair, with a hugely variable incidence from 2% to 31% [11]. For younger children with CoA/HAA, EEEA, EESA and APA are widely utilized techniques. EEEA could adequately relieve the stenosis of distal transverse arch and aortic isthmus while maintaining the arch physiologic geometry and avoiding the formation of Gothic arch [15]. Compared with EEEA, EESA can more effectively relieve proximal transverse arch stenosis. Rakhra *et al.* [16] described that patients undergoing end-to-side anastomosis via sternotomy had less arch reobstruction than those undergoing EEEA via thoracotomy, which might be explained by the above reason. For patients with severe HAA, recent studies [12, 17, 18] suggest autologous pulmonary patch repair seems to have fewer short-term complications, such as airway and pulmonary compression, and a lower incidence of reobstruction in mid-term follow-up. In our centre, EEEA is the first choice for patients with predominantly distal stenosis of a transverse arch. However, EEEA is not suitable for cases whose distal transverse arch is too long or extremely narrow. EESA is indicated in patients with an obvious proximal transverse arch stenosis or a long segmental stenosis in distal transverse arch. Autologous pericardial patch augmentation is almost abandoned because of the long-term calcification and lack of growth potential. We prefer autologous pulmonary patch augmentation in a patient with a severe HAA or a long gap between potential anastomotic sites. A long gap may cause high tension by direct tissue-to-tissue techniques (EEEA and EESA). It must be admitted that there is still no clear quantitative index for the selection of surgical techniques. To a certain extent, it depends on the surgeon's judgement on the specific arch anatomy and vascular tension after fully mobilizing the aorta and its branches. It is worth noting that there was no significant difference in early outcomes and long-term aortic reobstruction rates among the 3 groups. This suggests that there is no ‘best’ but ‘optimal’ surgical technique for varied aortic arch, and an appropriate choice can always bring a satisfied result.

### Limitations

Our study had limitations inherent in a retrospective study with small sample size. Only less half of the patients were followed up for >5 years. The results of this study need to be validated in multicentre studies with more patient numbers and longer follow-up.

## CONCLUSION

Aortic arch reconstruction in CoA/HAA can be successfully performed by EEEA, EESA and APA (autologous pulmonary patch augmentation) with satisfactory outcomes via median sternotomy. Preoperative aortic arch geometry study is helpful to select the appropriate surgical technique. The incidence of airway compression after arch repair is underestimated. CPB and postoperative LMBC detected by flexible fibre-optic bronchoscopy are significant predictors for long-term arch reobstruction.

## Supplementary Material

ivad186_Supplementary_Data

## Data Availability

The data underlying this article will be shared on reasonable request to the corresponding author.

## ABBREVIATIONS

ACC: Aortic cross-clamp
ACP: Antegrade cerebral perfusion
APA: Autograft patch augmentation
CI: Confidence interval
CoA: Coarctation of aorta
CoA/HAA: Coarctation of aorta with hypoplastic aortic arch
CPB: Cardiopulmonary bypass
DHCA: Deep hypothermic circulatory arrest
EEEA: Extended end-to-end anastomosis
EESA: Extended end-to-side anastomosis
HAA: Hypoplastic aortic arch
ICU: Intensive care unit
IQR: Interquartile range
LMBC: Left main bronchus compression
TTE: Transthoracic echocardiography
