# Supplementary material for: Bronchus compression is a predictor for reobstruction in coarctation with hypoplastic arch repair
Source: Interdiscip Cardiovasc Thorac Surg. 2023 Nov 22;37(5):ivad186. doi: 10.1093/icvts/ivad186 (PMC10681811; doi:10.1093/icvts/ivad186)
Supplement: ivad186_Supplementary_Data [file ivad186_supplementary_data.zip › Supplemental figure legend and tables.docx]

**SUPPLEMENTAL FIGURE 1.** (A) Schematic incisions of three different surgical techniques. Red dotted line：extended end-to-end anastomosis；Blue dotted line: extended end-to-side anastomosis; Green dotted line: autograft patch augmentation. (B) Aortic arch after extended end-to-end anastomosis. (C) Aortic arch after extended end-to-side anastomosis. (D) Aortic arch after autograft patch augmentation.

**SUPPLEMENTAL FIGURE 2.** Kaplan-Meier curves depicting freedom from reobstruction for 3 groups. The shade area represents the 95% confidence interval. The Mantel-Cox log-rank test showed no significant difference in reobstruction rates among the three groups (P=0.33).

**SUPPLEMENTAL TABLE 1.** Concomitant procedures

| **Variable n(%)** | **Overall**  **(n=104)** | **EEEA**  **(n=41)** | **EESA**  **(n=38）** | **APA**  **(n=25)** |
| --- | --- | --- | --- | --- |
| Atrial septal defect closure | 89 (85.6) | 36 (87.8) | 30 (78.9) | 23 (92.0) |
| Ventricular septal defect closure | 87 (83.7) | 32 (78.0) | 32 (84.2) | 23 (92.0) |
| Mitral valve repair | 7 (6.7) | 4 (9.8) | 3 (7.9) | 0 (0) |
| Aortic stenosis repair |  |  |  |  |
| Subvalvular myectomy | 2 (1.9) | 1 (2.4) | 1 (2.6) | 0 (0) |
| Valvulotomy | 1 (1.0) | 1 (2.4) | 0 (0) | 0 (0) |
| Tricuspid valve repair | 3 (2.9) | 2 (4.9) | 0 (0) | 1 (4.0) |
| Partial anomalous pulmonary venous connection repair | 1 (1.0) | 0 (0) | 1 (2.6) | 0 (0) |
| Right ventricular outflow tract reconstruction | 1 (1.0) | 1 (2.4) | 0 (0) | 0 (0) |
| Double-outlet right ventricle repair | 1 (1.0) | 1 (2.4) | 0 (0) | 0(0) |
| Arterial switch operation | 1 (1.0) | 0 (0) | 0 (0) | 1 (4.0) |

APA, autograft patch augmentation; EEEA, extended end-to-end anastomosis; EESA, extended end-to-side anastomosis.

**SUPPLEMENTAL TABLE 2.** The patient characteristics, preoperative aortic arch geometry and main recovery indices in patients with and without postoperative left main bronchus compression

|  | **Left Main Bronchus Compression** | | **P** |
| --- | --- | --- | --- |
|  | **No (n=85)** | **Yes (n=19)** |  |
| Age at surgery, days, median (IQR) | 43.0 (13.5 to 93.5) | 36.0 (17.0 to 67.0) | 0.509 |
| Weight at surgery, kg, median (IQR) | 3.80 (3.30 to 4.90) | 3.40 (3.0 to 4.0) | 0.046 |
| Preoperative PGE1 use, n (%) | 10 (11.8) | 7 (36.8%) | 0.008 |
| Proximal transverse arch z score, median (IQR) | -2.53 (-3.86 to -1.90) | -2.74 (-3.67 to -1.98) | 0.950 |
| Proximal transverse arch length, mm, median (IQR) | 2.1 (0 to 2.9) | 2.3 (1.3 to 3.7) | 0.519 |
| Distal transverse arch z score, median (IQR) | -3.04 (-4.26 to -2.37) | -3.22 (-3.77 to 2.68) | 0.900 |
| Distal transverse arch length, mm, median (IQR) | 6.2 (3.6 to 8.2) | 4.9 (2.6 to 8.7) | 0.507 |
| Aortic isthmic z score, median (IQR) | -3.94 (-5.00 to -2.90) | -3.55 (-5.14 to -2.17) | 0.449 |
| Bovine aortic arch, n (%) | 25 (29.4) | 4 (21.1) | 0.463 |
| Postoperative mechanical ventilation time, hours, median (IQR) | 97.8 (72.4 to 149.1) | 122.5 (98.0 to 150.0) | 0.106 |
| Postoperative ICU length of stay, days, median (IQR) | 15.0 (10.0 to 19.8) | 21.0 (13.0 to 23.0) | 0.037 |
| Hospital length of stay, days, median (IQR) | 23.0 (19.0 to 28.0) | 27.0 (25.0 to 34.0) | 0.002 |

ICU, intensive care unit; IQR, interquartile range.
