# Supplementary figures and images for: Bronchus compression is a predictor for reobstruction in coarctation with hypoplastic arch repair
Source: Interdiscip Cardiovasc Thorac Surg. 2023 Nov 22;37(5):ivad186. doi: 10.1093/icvts/ivad186 (PMC10681811; doi:10.1093/icvts/ivad186)

**A**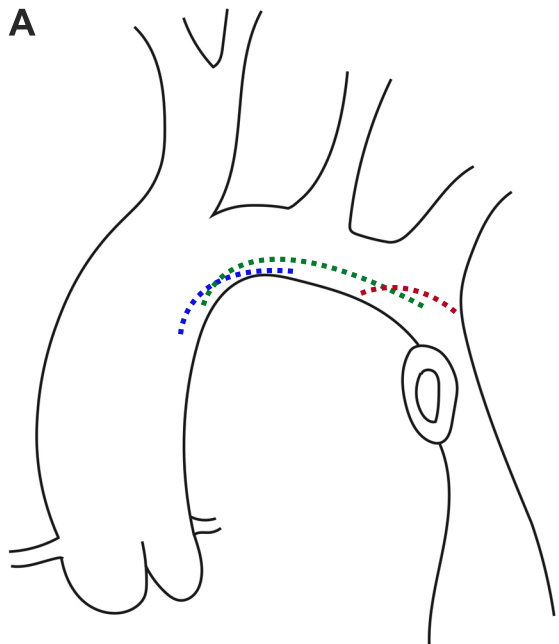**B**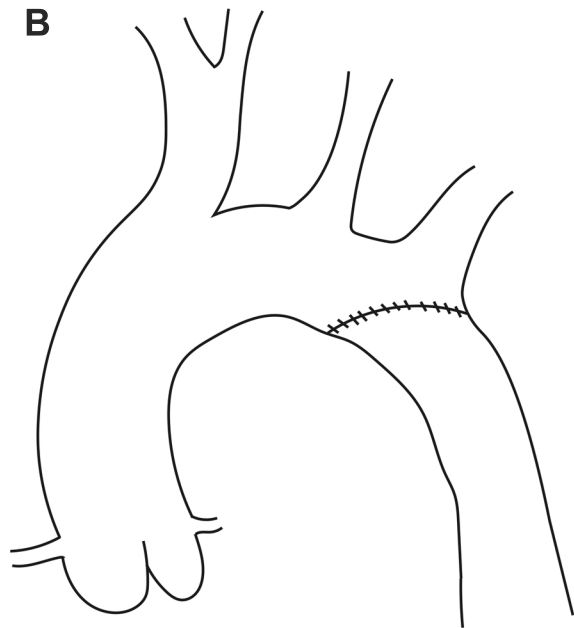**C**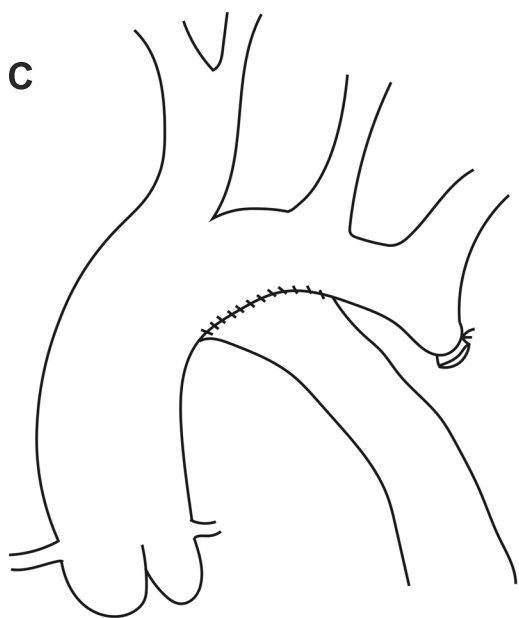**D**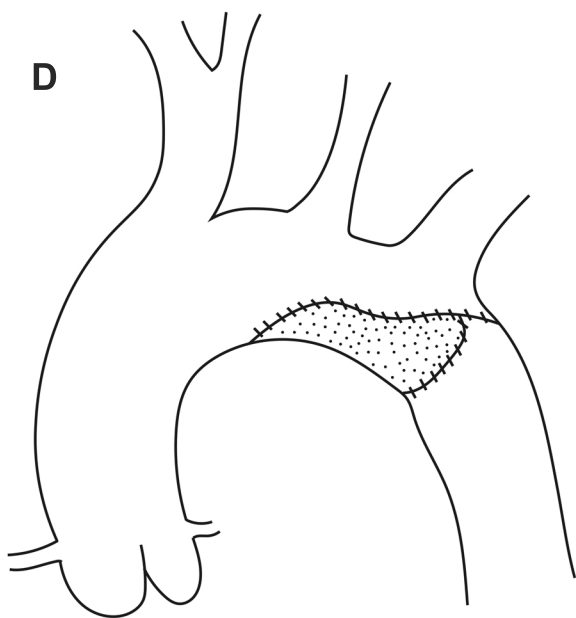

Supplement: ivad186_Supplementary_Data [file ivad186_supplementary_data.zip › Supplemental Figure1.pdf]

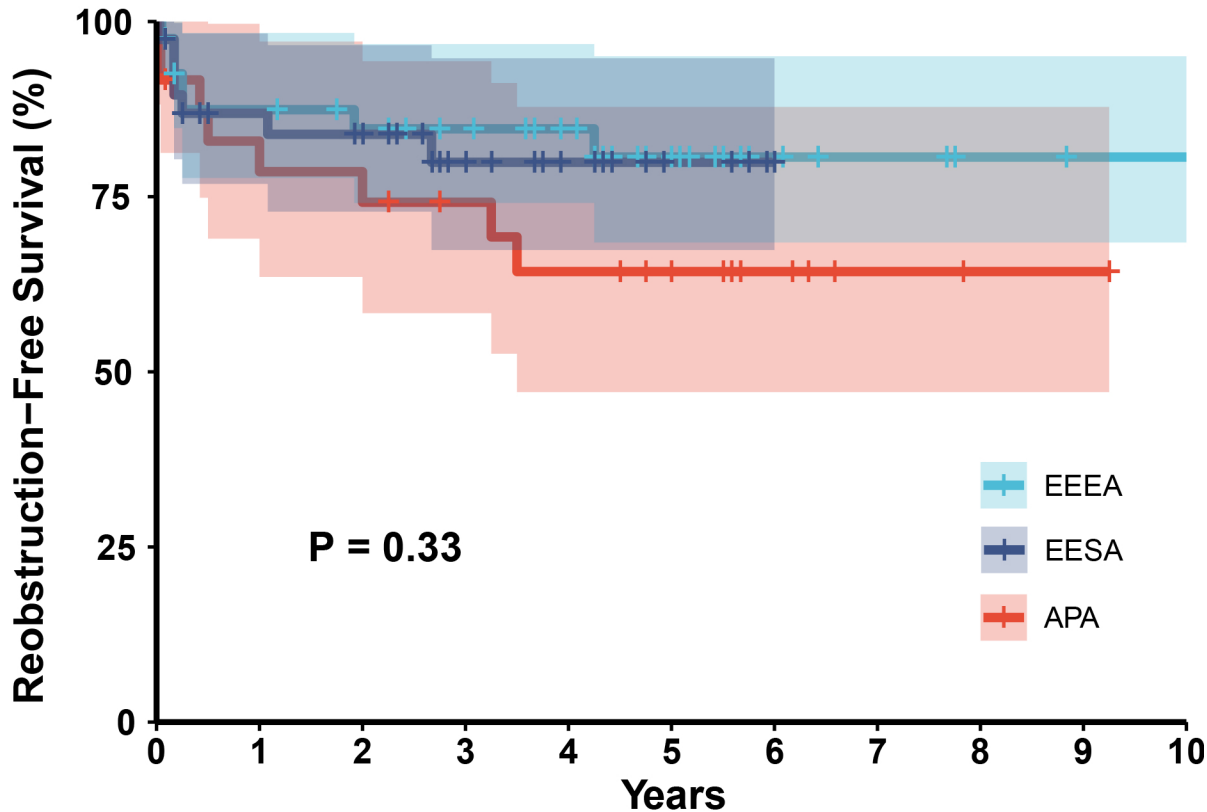

### Patients at risk

|       |    |    |    |    |    |    |   |
|-------|----|----|----|----|----|----|---|
| EEEEA | 41 | 34 | 31 | 27 | 23 | 14 | 6 |
| EESA  | 38 | 29 | 27 | 17 | 13 | 5  | 1 |
| APA   | 25 | 19 | 18 | 15 | 13 | 11 | 6 |

Supplement: ivad186_Supplementary_Data [file ivad186_supplementary_data.zip › Supplemental Figure2.pdf]
